# Supplementary material for: Single Cell Transcriptomic and Chromatin Profiles Suggest Layer Vb Is the Only Layer With Shared Excitatory Cell Types in the Medial and Lateral Entorhinal Cortex
Source: Front Neural Circuits. 2022 Jan 26;15:806154. doi: 10.3389/fncir.2021.806154 (PMC8826650; doi:10.3389/fncir.2021.806154)
Supplement: Supplementary file 14 [file Data_Sheet_1.ZIP › LEC_Sup_vs_MEC_Sup_Motifs/homerResults/motif5.similar.html]

motif5

## Information for motif5

A
C
T
G
A
C
T
G
A
C
T
G
A
C
T
G
A
C
T
G
A
C
T
G
A
C
T
G
A
C
T
G
A
C
T
G
A
C
T
G
  
Reverse Opposite:  

A
G
T
C
A
G
T
C
A
G
T
C
A
G
T
C
A
G
T
C
A
G
T
C
A
G
T
C
A
G
T
C
A
G
T
C
A
G
T
C
  

|  |  |
| --- | --- |
| p-value: | 1e-50 |
| log p-value: | -1.164e+02 |
| Information Content per bp: | 1.530 |
| Number of Target Sequences with motif | 4108.0 |
| Percentage of Target Sequences with motif | 30.81% |
| Number of Background Sequences with motif | 4989.6 |
| Percentage of Background Sequences with motif | 25.04% |
| Average Position of motif in Targets | 359.9 +/- 252.8bp |
| Average Position of motif in Background | 386.2 +/- 260.4bp |
| Strand Bias (log2 ratio + to - strand density) | -0.1 |
| Multiplicity (# of sites on avg that occur together) | 1.53 |
| Motif File: | file (matrix) reverse opposite |

### Similar de novo motifs found

|  |  |  |  |  |  |  |  |
| --- | --- | --- | --- | --- | --- | --- | --- |
| Rank | Match Score | Redundant Motif | P-value | log P-value | % of Targets | % of Background | Motif file |
| 1 | 0.628 | G A T C C T A G A T G C G T A C C A T G A T G C G T A C C A T G A G T C G A T C | 1e-8 | -18.783567 | 1.09% | 0.66% | motif file (matrix) |
| 2 | 0.639 | C G A T G T A C A T G C G A T C A G C T T A G C G T A C A T G C A C T G A T G C | 1e0 | -2.130307 | 2.56% | 2.41% | motif file (matrix) |
